# Comprehensive Analysis of Inflammatory Response–Related Genes, and Prognosis and Immune Infiltration in Patients With Low-Grade Glioma

**DOI:** 10.3389/fphar.2021.748993

**Published:** 2021-10-12

**Authors:** Tao Han, Zhifan Zuo, Meilin Qu, Yinghui Zhou, Qing Li, Hongjin Wang

**Affiliations:** ^1^ Department of Oncology, The First Affiliated Hospital of China Medical University, Shenyang, China; ^2^ The General Hospital of Northern Theater Command Training Base for Graduate, China Medical University, Shenyang, China; ^3^ School of Life Science and Biopharmaceutics, Shenyang Pharmaceutical University, Shenyang, China; ^4^ The General Hospital of Northern Theater Command Training Base for Graduate, Jinzhou Medical University, Jinzhou, China; ^5^ School of Pharmacy, Shenyang Pharmaceutical University, Shenyang, China; ^6^ Department of Neurology, The Second Affiliated Hospital of Dalian Medical University, Dalian, China

**Keywords:** low-grade glioma, inflammatory response, prognosis, immune infiltration, drug sensitivity

## Abstract

**Background:** Although low-grade glioma (LGG) has a good prognosis, it is prone to malignant transformation into high-grade glioma. It has been confirmed that the characteristics of inflammatory factors and immune microenvironment are closely related to the occurrence and development of tumors. It is necessary to clarify the role of inflammatory genes and immune infiltration in LGG.

**Methods:** We downloaded the transcriptome gene expression data and corresponding clinical data of LGG patients from the TCGA and GTEX databases to screen prognosis-related differentially expressed inflammatory genes with the difference analysis and single-factor Cox regression analysis. The prognostic risk model was constructed by LASSO Cox regression analysis, which enables us to compare the overall survival rate of high- and low-risk groups in the model by Kaplan–Meier analysis and subsequently draw the risk curve and survival status diagram. We analyzed the accuracy of the prediction model *via* ROC curves and performed GSEA enrichment analysis. The ssGSEA algorithm was used to calculate the score of immune cell infiltration and the activity of immune-related pathways. The CellMiner database was used to study drug sensitivity.

**Results:** In this study, 3 genes (CALCRL, MMP14, and SELL) were selected from 9 prognosis-related differential inflammation genes through LASSO Cox regression analysis to construct a prognostic risk model. Further analysis showed that the risk score was negatively correlated with the prognosis, and the ROC curve showed that the accuracy of the model was better. The age, grade, and risk score can be used as independent prognostic factors (*p* < 0.001). GSEA analysis confirmed that 6 immune-related pathways were enriched in the high-risk group. We found that the degree of infiltration of 12 immune cell subpopulations and the scores of 13 immune functions and pathways in the high-risk group were significantly increased by applying the ssGSEA method (*p* < 0.05). Finally, we explored the relationship between the genes in the model and the susceptibility of drugs.

**Conclusion:** This study analyzed the correlation between the inflammation-related risk model and the immune microenvironment. It is expected to provide a reference for the screening of LGG prognostic markers and the evaluation of immune response.

## Introduction

Glioma is the most common primary malignant tumor of the central nervous system, and its rate of fatality and disability are both high (Lapointe et al., 2018; Ferlay et al., 2019). Low-grade glioma is classified into grade II and grade III with isocitrate dehydrogenase (IDH) mutations according to the WHO histopathological grading system (De Blank et al., 2020). Although its prognosis is relatively better than that of high-grade gliomas, nearly 70% of LGG patients, during the period of occurrence and development, are prone to transition to high-grade gliomas (HGGs), which express the characteristics such as higher malignant degree and stronger invasiveness (Appolloni et al., 2019; Huang, 2019; Nejo et al., 2019; Youssef and Miller, 2020). At present, the main clinical treatments for LGG include surgical resection, radiotherapy, and chemotherapy. However, the existing treatments still fail to significantly improve the survival rate of patients. It is well known that cancer is closely related to inflammation. Rudolf Virchow et al. regarded inflammation as a possible driver of tumorigenesis through “lymphatic reticular infiltration,” and the inflammatory cells and cytokines in tumor contributed to the growth, progression, and immunosuppression of cancer (Bergamin et al., 2015; Lepore et al., 2018). Inflammation is one of the characteristics of tumors, and uncontrollable inflammation is closely related to the occurrence, development, invasion, and metastasis of tumors (Singh et al., 2017). The growth of tumors depends not only on the genetic changes of malignant tumor cells but also on the changes in the tumor microenvironment, such as matrix, blood vessels, and infiltrating inflammatory cells, and it is immunity and inflammation that constitute the two cores of the tumor microenvironment (Chen and Mellman, 2017; Suarez-Carmona et al., 2017). In recent years, increasing evidence has shown that tumor-related inflammation can advance tumor growth and progression by promoting angiogenesis and metastasis, subverting antitumor immune responses, and changing the sensitivity of tumor cells to chemotherapeutics (Khandia and Munjal, 2020). Cytokines, which are produced by chronic inflammation, induce gene mutations, change the expression and transformation of oncogenes and tumor suppressor genes, inhibit cell apoptosis, evoke angiogenesis, and result in abnormal inflammatory signal transduction pathways. In addition, chronic inflammation can recruit a variety of immunosuppressive cells (such as M2-TAMs, MDSC, and Treg) to facilitate the establishment of immunosuppressive tumor microenvironment and accelerate the occurrence of the tumor malignant biological behavior (Whiteside, 2018; Zhang et al., 2019; Weber et al., 2021). Therefore, it is of great significance to effectively control chronic inflammation in the process of inhibiting tumor occurrence and enhancing antitumor immune response. It is this idea that is the starting point of this article to carry out research, hoping to provide a certain reference for related research on tumor inflammation and immunity.

Some studies have shown that inflammatory media, which contain human leukocyte antigen-G, CD8 T cells, IL-1beta, IL-6, TAM, the S100A family and so on, are having high expression in the high-risk group (Bloch et al., 2013; Kelly et al., 2020). It is seen that the proliferation, deterioration, and prognosis of glioma are closely linked with the inflammatory microenvironment. Currently, some inflammatory response–related genes were reported to predict the metastasis potential of LGG, but further utilization in the prognosis of LGG remains to be studied. In addition, the integrity of the blood–brain barrier of glioma is easily destroyed with the help of pathological conditions, which provide opportunities for immune-related cell infiltration and recognition (Cserr et al., 1992; Davies, 2002; Goldmann et al., 2006). It has laid a theoretical foundation for developing immunotherapy in LGG. Therefore, it is necessary to establish an inflammatory factor–related model derived from LGG samples to scientifically predict its prognosis.

In this study, we downloaded the transcriptome gene expression data and corresponding clinical data of LGG patients from public databases. Then, we constructed a prognostic model using differentially expressed genes related to inflammation in the TCGA database, and verified the accuracy of the model in predicting the survival of LGG patients through the ROC curve. Then, we further conducted a functional enrichment analysis to explore its potential immune mechanism. In addition, we also analyzed the relationship between the prognostic gene expression and the type of immune infiltration, and discussed the feasibility of inflammation-related risk models in predicting the immunotherapy response. Finally, we analyzed the inflammatory genes in the prognosis model and the sensitivity of drugs. Our research has discovered that some inflammation-related genes may act as early warning markers and were considered to be related to the poor prognosis of LGG, and meanwhile, we also clarified its relevance to the immune microenvironment, which may provide an important basis for the evaluation of the clinical efficacy of immunotherapy.

## Methods

### Data Acquisition Extract

We downloaded transcriptional group gene expression data from the tumor samples of 529 patients with LGG through the Cancer Genome Atlas (TCGA, https://portal.gdc.cancer.gov/) database while obtaining the corresponding clinical information, including age, gender, grade, overall survival time, and survival status. We downloaded the gene expression data of 1,152 normal brain tissues in the GTEX database through the UCSC website (http://xena.ucsc.edu/). We extracted and verified the reaction-related genes: downloaded the human inflammatory response gene set from the GSEA (http://www.gsea-msigdb.org/gsea/index.jsp) database (Supplementary Tables 6, 7, and 9).

### Screening of Differential Genes and Prognosis-Related Inflammation Genes

In order to obtain differentially expressed inflammation-related genes, we used the “Bioconductor Limma” R package to analyze 529 LGG tumor tissues and 1,152 normal brain tissues. If a gene satisfies the condition of | log2FC | > 2 and FDR <0.05, it was considered to be a differential inflammatory gene. In order to clarify the relationship between inflammation-related genes and the prognosis of LGG patients, based on the expression data and clinical data of tumor samples in the TCGA database, we used the “survival” R package to perform univariate Cox regression analysis on inflammation genes. A gene was considered to be a prognosis-related gene if *p* < 0.05.

### Construction and Evaluation of Inflammatory Gene–Related Prognostic Models

In order to determine the value of inflammatory genes in evaluating the prognosis of LGG, we adopted LASSO Cox regression analysis to construct a prognostic risk model which employed the abovementioned nine prognosis-related differential inflammatory genes. The risk score is calculated using the following formula: Risk score = $\sum_{i=1}^{n}\text{Coe}f_{\text{i}}*x_{\text{i}}$ , In this formula, Coefi is the risk coefficient and xi is the expression of each gene. According to the median risk score, LGG patients were divided into the high-risk group and low-risk group, meanwhile drawing the risk curve and survival status chart. We employed PCA analysis by using the “ggplot2”R package to explore the distribution of genes in different groups based on the expression level of genes in the model.

Analyzing the prognostic value of the model that enables us to exploit the “survival” package and “survminer” R package to analyze the relationship between patients with different risk groups and overall survival, the survival curve was drawn. The “timeROC” R package was used to construct the receiver operating characteristic (ROC) curve to evaluate prediction efficiency. Besides, we eliminated samples with incomplete clinical information in the TCGA database, and utilized univariate and multivariate Cox regression analyses to explore the feasibility of prognostic risk models as independent prognostic markers. Finally, the relationship of LGG patients’ age, gender, and classification between the risk score of the prognostic model was clarified by means of clinical correlation analysis.

### GSEA Enrichment Analysis

To illuminate the enrichment of high- and low-risk LGG sample groups in terms of the immune function, the study used gene set enrichment analysis (GSEA) and GSEA 4.1.0 software to carry out Genome Encyclopedia (KEGG) pathway enrichment analysis. We believe that when *p* < 0.05, this pathway is considered statistically significant.

### Comprehensive Analysis of Tumor Microenvironment and Tumor Immune Correlation

With the purpose of clarifying the correlation between the inflammatory response and immune infiltration, our team first adopted the tumor immune cell infiltration score and immune-related function score for each LGG sample in the TCGA database by applying the ssGSEA method and sequentially used the “Bioconductor Limma” R package to operate the differential analysis of immune scoring and immune typing, and drew the box plot. We used the Spearman correlation test to evaluate the risk score to explore the relationship between the expression of immune checkpoints such as PD-1 and PD-L1 and the stem cell index (DNA-based DNA methylation and RNA-based RNA expression). Next, we performed an analysis of the immune score, matrix score, and comprehensive score on LGG samples in the TCGA database, using the “estimate” R package and the “Limma” R package to export a scatter chart.

### Drug Sensitivity Analysis

With an aim to clarify the influence of inflammatory genes in the prognostic model on drug sensitivity and tolerance, we downloaded transcriptome data from the CellMiner database (https://discover.nci.nih.gov/cellminer/) and FDA-certified drug sensitivity–related data. The Pearson correlation test was utilized to analyze the relationship between gene expression and drug sensitivity. Next, we used the “pRRophetic” R package to analyze the relationship between the high- and low-risk groups in the prognosis model and LGG-related drugs, and draw a box plot.

### Statistical Analysis

We adopt the Wilcoxon rank-sum test to analyze the gene differences between tumor tissues and normal tissues. Our group had taken the method of the LASSO Cox regression algorithm to establish a risk prognosis model, wherein the relationship between its overall survival rate in the high- and low-risk group gene expression group was used to generate the Kaplan–Meier survival curve, and the accuracy of the model was tested by the ROC curve. Univariate and multivariate Cox regression analyses were used to evaluate the feasibility of the risk score as an independent prognostic factor. The chi-square test was used to compare the differences of clinical traits between different risk groups. We used two correlation test means. One is Spearman's test which analyzed the relationship between the sample risk score and the expression of immune checkpoints, such as PD-1 and PD-L1, stem cell index, and the tumor microenvironment score. The other is Pearson's test which evaluated the correlation between the gene expression and drug sensitivity in the model. All above statistical analyses were performed using R (version 4.0.4) software. If *p* < 0.05, it was considered statistically significant.

## Results

### Screening of Prognosis-Related Differential Inflammation Genes

**FIGURE 1:**
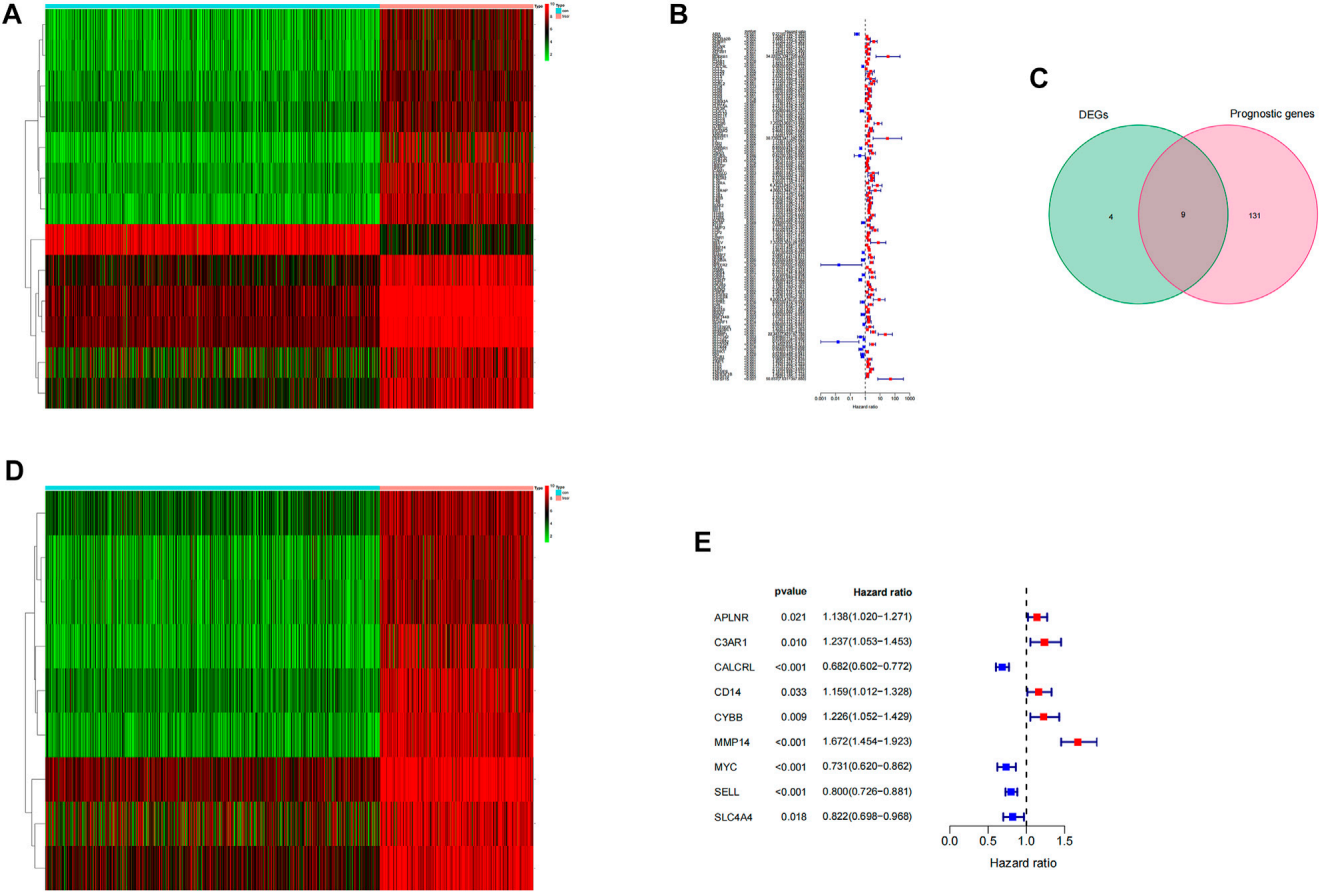
Screening of prognostic differential inflammatory genes. **(A)** Thermal map of all inflammatory differential gene expression in LGG tumor tissues and normal tissues. **(B)** Based on 200 inflammatory genes, the forest map of prognosis-related genes was screened.**(C)** Wayne diagram of intersection of differential genes and prognosis-related genes. **(D)** Thermograms of nine prognosis-related inflammatory genes. **(E)** Forest map of nine prognosis-related inflammatory genes.

We downloaded the human inflammatory response gene set using the GSEA database, which contained 200 inflammatory response–related genes, such as ABCA1, ABI1, and ACVR1 B. The expression levels of these inflammatory genes in LGG tissues and normal brain tissues were obtained from the TCGA database and GTEX database, and 13 differentially expressed inflammatory genes were screened. Compared with normal tissues, ABCA1, APLNR, BTG2, C3AR1, CALCRL, CD14, CYBB, HIF1A, MMP14, MYC, SELL, and SLC4A4 were upregulated in LGG tumor tissues, and SCN1B was downregulated in tumor tissues (Figure 1A). Then, we used the expression data and clinical information of LGG samples in the TCGA database for univariate COX analysis. A total of 140 inflammatory genes related to prognosis were obtained, including 21 low-risk genes and 119 high-risk genes (Figure 1B). Finally, we crossed the differentially expressed genes with prognosis-related genes and obtained 9 inflammation-related genes that can mediate prognosis and have differential expression in LGG patients (Figures 1C–E).

### Construction and Evaluation of a Prognostic Model of Inflammation-Related Genes

**FIGURE 2:**
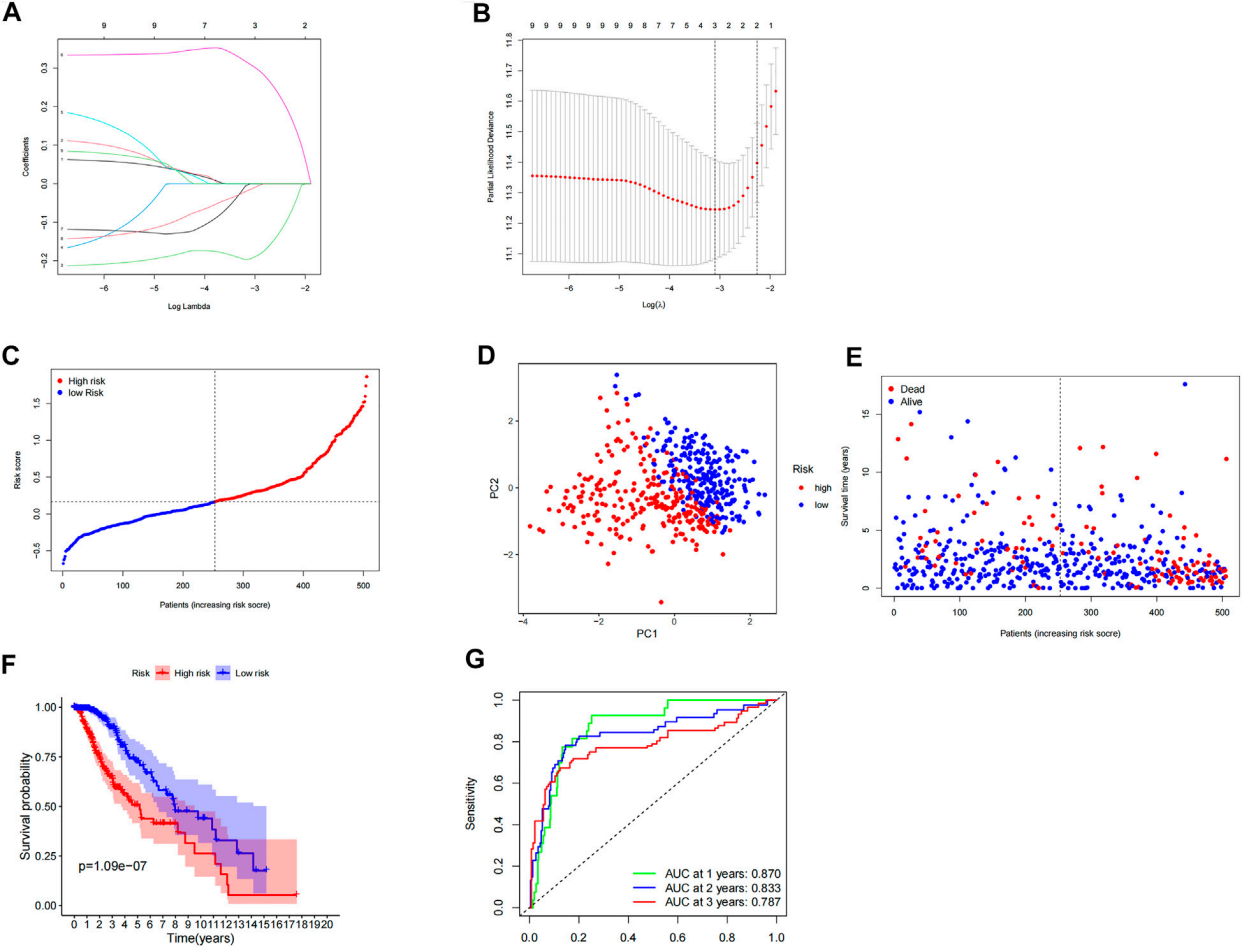
Construction and evaluation of prognostic model of inflammation-related genes. **(A,B)** LASSO Cox regression analysis screened three differentially expressed inflammatory genes and established a prognostic model. **(C)** Risk curve constructed according to the median of the risk score. **(D)** Feasibility of PCA-based analysis and judgment models. **(E)** Survival status of LGG patients with different risk scores. **(F)** Survival analysis of LGG patients in different risk groups.**(G)** ROC curve suggested that the model had good accuracy in predicting the 1-year, 3-year, and 5-year survival of LGG patients.

We used LASSO Cox regression analysis to analyze the significant prognostic differential inflammatory genes in the abovementioned nine univariate results, and finally identified three genes (CALCRL, MMP14, and SELL) for the construction of prognostic risk models (Figures 2A,B). At the same time, the weight coefficient of each gene was determined, and the risk score was calculated according to the following formula: Risk score = -0.195793×CALCRL+0.310011×MMP14-0.015233×SELL.

According to the median risk score, LGG patients were divided into high- and low-risk groups (Figure 2C). PCA analysis showed that patients with different risk groups were distributed in two directions, indicating that the expression of three genes in the model can effectively classify LGG patients into high- and low-risk groups (Figure 2D). The survival status scatterplot showed that the number of patients who died gradually increased with the increase in the risk value (Figure 2E). Further survival analysis showed that the overall survival time was significantly different between the high-risk group and the low-risk group, and the overall survival rate of the high-risk group was significantly lower than that of the low-risk group (*p* < 0.001) (Figure 2F). The ROC curve showed that the AUC values of the model for 1 year, 3 years, and 5 years were 0.870, 0.833, and 0.787, respectively, indicating that the model had high accuracy in predicting the survival of LGG patients (Figure 2G).

### Independent Prognostic Analysis and Clinical Correlation Analysis

**FIGURE 3:**
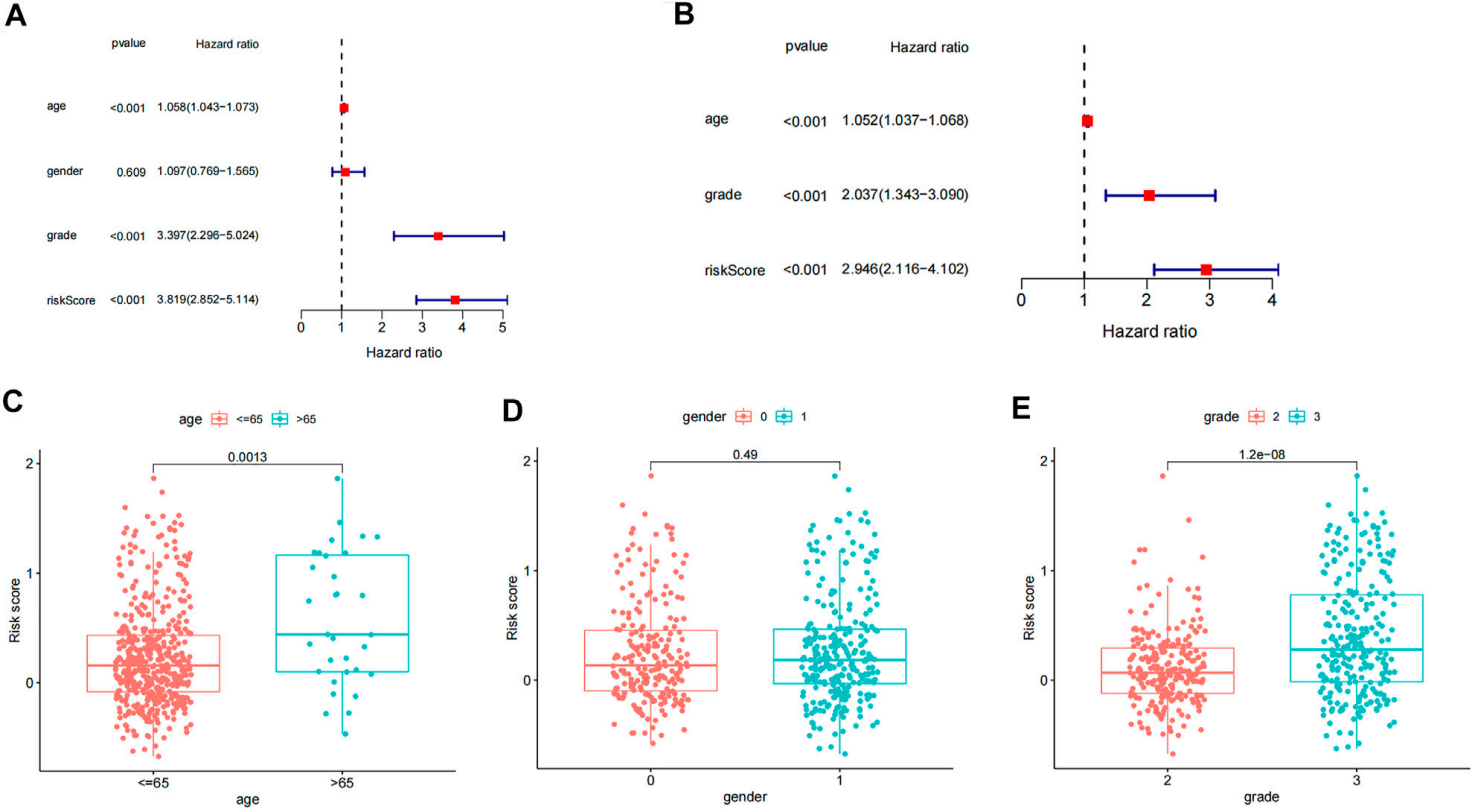
Independent prognostic analysis and clinical correlation analysis. **(A)** Single-factor Cox regression analysis of different clinical characteristics and risk scores. **(B)** Multivariate Cox regression analysis of different clinical characteristics and risk scores. **(C–E)** Differences in age, gender, and tumor grade between high- and low-risk groups.

We explored the independent prognostic value of the inflammation-related gene prognostic risk model through single-factor regression analysis and multifactor regression analysis. Univariate analysis showed that age (*p* < 0.001), grading (*p* < 0.001), and risk score (*p* < 0.001) were significantly correlated with the overall survival rate of patients (Figure 3A). Further multivariate analysis showed that age (*p* < 0.001), grading (*p* < 0.001), and risk score (*p* < 0.001) were still significantly correlated with the overall survival rate of patients (Figure 3B). Therefore, we believe that the risk score of the model can be used as an independent prognostic factor for LGG. By analyzing the relationship between the risk score and clinical characteristics, we found that the risk score of LGG patients aged ≥65 years was significantly higher than that of patients aged <65 years (*p* < 0.01), and the risk score of patients with tumor grade 3 was significantly higher. For grade 2 patients (*p* < 0.001), there was no significant difference in risk scores between different genders (*p* > 0.05) (Figures 3C–E).

### Pathway Enrichment Analysis

**FIGURE 4:**
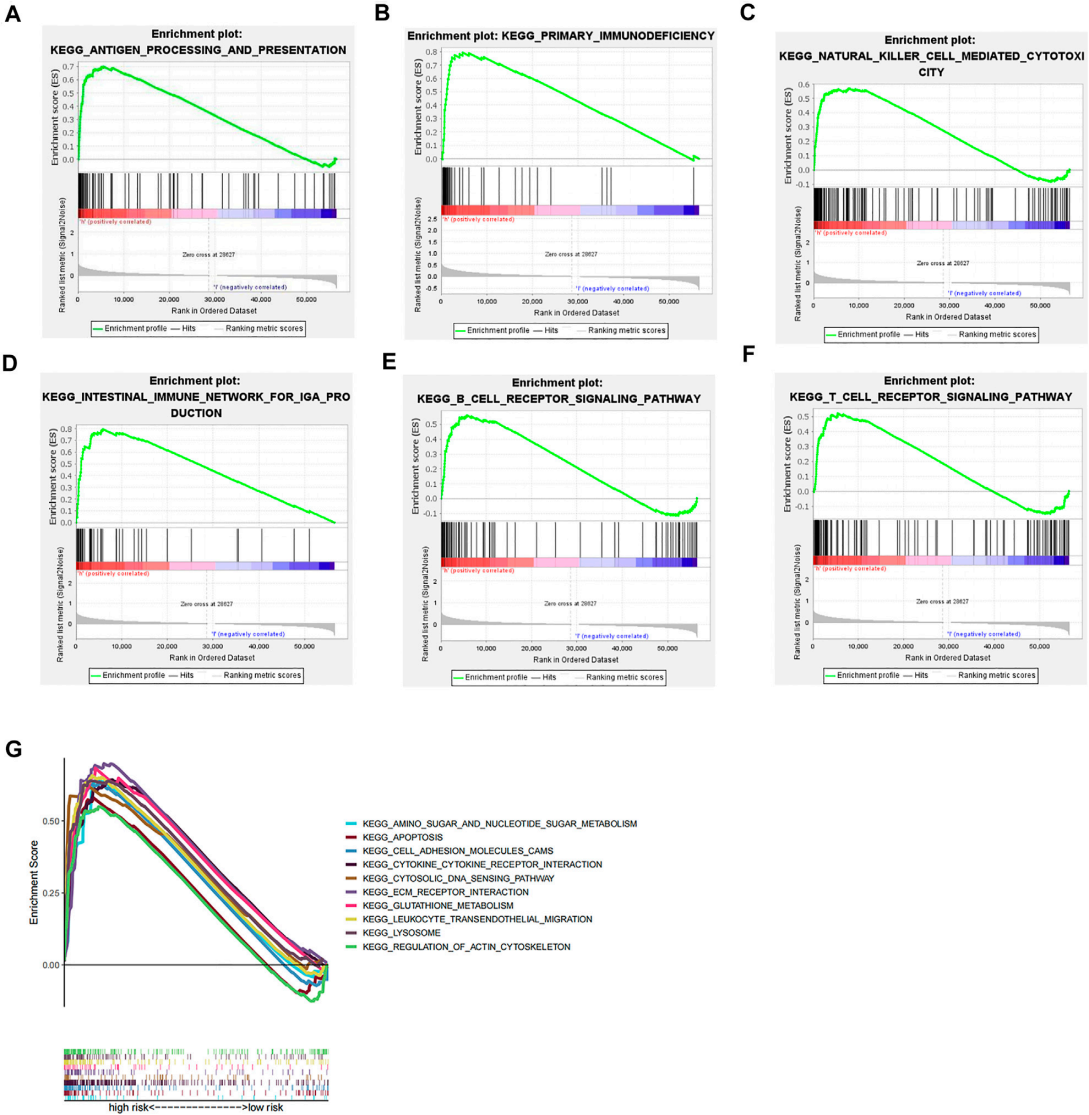
GSEA pathway enrichment analysis. **(A–F)** Immune-related pathways that are significantly enriched in the high-risk group. **(G)** Top ten pathways for GSEA enrichment analysis.

We performed GSEA pathway enrichment analysis on the high- and low-risk groups, and the results showed that 64 pathways were significantly enriched in the high-risk group (false discovery rate <0.05). Among them, the statistically prominent pathways include leukocyte transendothelial migration, glutathione metabolism, regulation of actin cytoskeleton, and apoptosis (Figure 4G). Further, we conducted an in-depth analysis of immune-related pathways, and the results indicate that the immune-related pathways of this model include antigen processing and presentation, primary immunodeficiency, natural killer cell–mediated cytotoxicity, intestinal immune IGA production network, and B-cell receptor body signaling pathway and T-cell receptor signaling pathway, but failure to find statistically significant pathways was enriched in the low-risk group (Figures 4A–F).

### Correlation Analysis of Immune Subtypes and Immune Response

**FIGURE 5:**
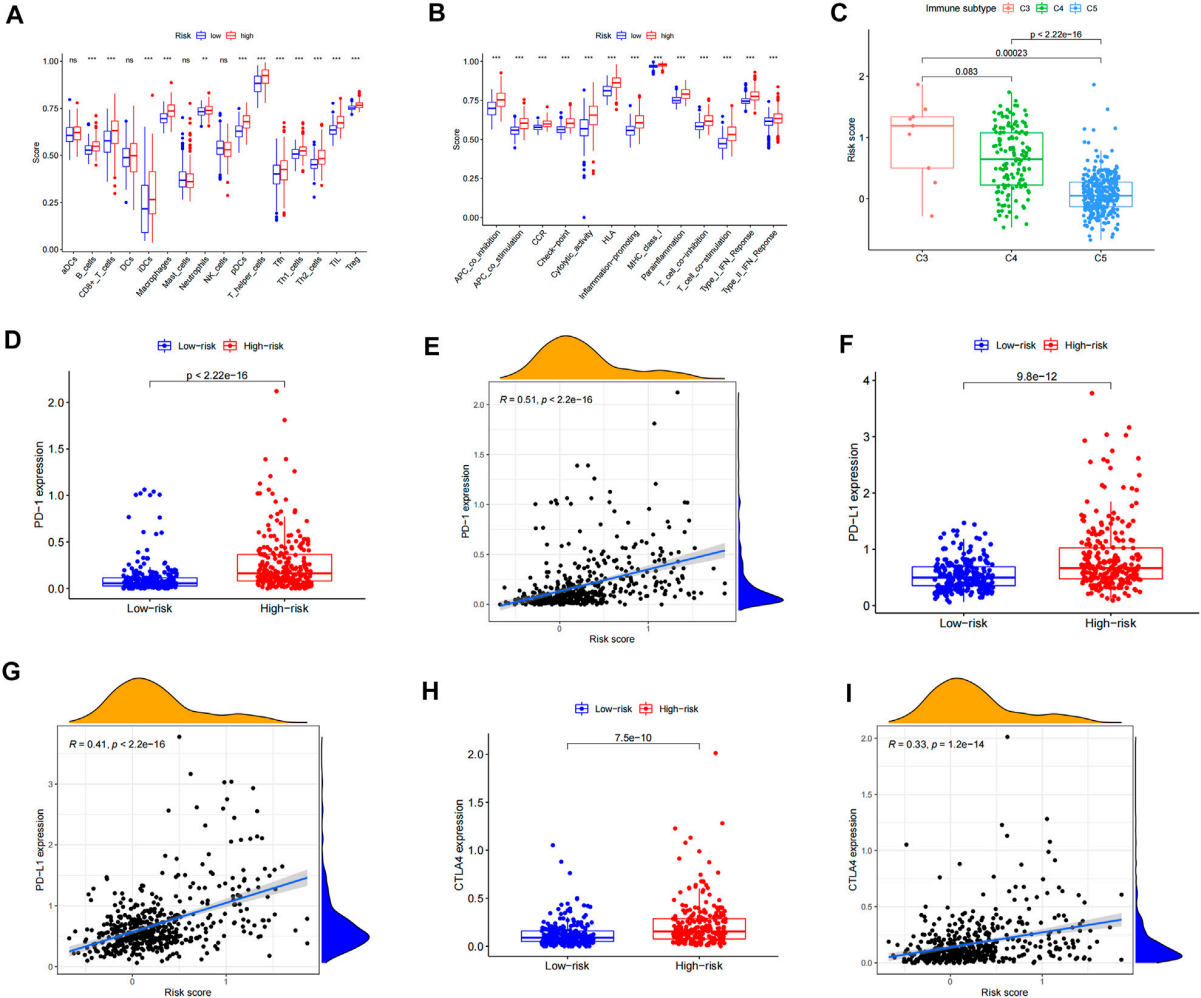
Correlation analysis of the immune infiltration pattern. **(A)** Differences of immune cell subsets in high- and low-risk groups of an inflammation-related prognosis model. **(B)** The difference of the immune function and pathway in the high- and low-risk groups of inflammation-related prognosis models. **(C)** The difference between LGG patients with different risk values and immune classification. **(D)** The expression difference of PD-1 in LGG high- and low-risk groups. **(E)** Scatterplot of PD-1 correlation with the risk score. **(F)** Expression difference of PD-L1 in LGG high- and low-risk groups. **(G)** Scatterplot of PD-L1 correlation with the risk score. **(H)** The expression difference of CTLA4 in LGG high- and low-risk groups. **(I)** Scatterplot of CTLA4 correlation with the risk score.

We used the ssGSEA method to quantify 16 immune cell subsets and 13 immune-related functions to clarify the correlation between the risk score and immune status. We found that the infiltration of 12 immune cell subsets in the high-risk group was significantly more than that of the low-risk group (*p* < 0.05), including B-cells, CD8 + T cells, iDCs, pDCs, macrophages, neutrophils, T-helper cells, Tfh, Th1 cells, Th2 cells, TIL, and Tregs (Figure 5A, Supplementary Tables 6, 7, and 9). Further analysis showed that compared with the low-risk group, the scores of 13 immune functions and pathways, including APC co-inhibition, APC co-stimulation, check point, and T-cell co-inhibition, were significantly higher in the high-risk group (*p* < 0.05) (Figure 5B). From the abovementioned results, we can see that the immune response is more active in the high-risk group than in the low-risk group, and the poor prognosis of LGG patients in the high-risk group may be related to negative immune regulation. In order to assess the differences between LGG patients with different risk values and immunophenotyping, according to the distribution of immunophenotyping of different types of tumor samples in the TCGA database, we combined inflammatory (Immune C3), lymphocyte-depleted (Immune C4), and immunologically quiet (Immune C5) responses. Three types were included in the LGG study, and the results showed that there were significant differences between C3 and C5, and C4 and C5 (*p* < 0.05), and Immune C3 had the largest risk score for LGG patients, whereas considering Immune C5 in LGG patients, the risk was minimal (Figure 5C). In order to evaluate the feasibility of the inflammation-related risk model in predicting the response of immunotherapy, we conducted a correlation study between the risk score and three inhibitory immune checkpoints. The results showed that the expressions of PD-1, PD-L1, and CTLA4 were significantly upregulated in the high-risk group relative to the low-risk group, and the expressions of PD-1, PD-L1, and CTLA4 were positively correlated with the risk score (*p* < 0.05) (Figures 5D–I). Therefore, the abovementioned results indicate that patients in the high-risk group can benefit from immunotherapy more clinically.

### Correlation Analysis of Tumor Microenvironment

**FIGURE 6:**
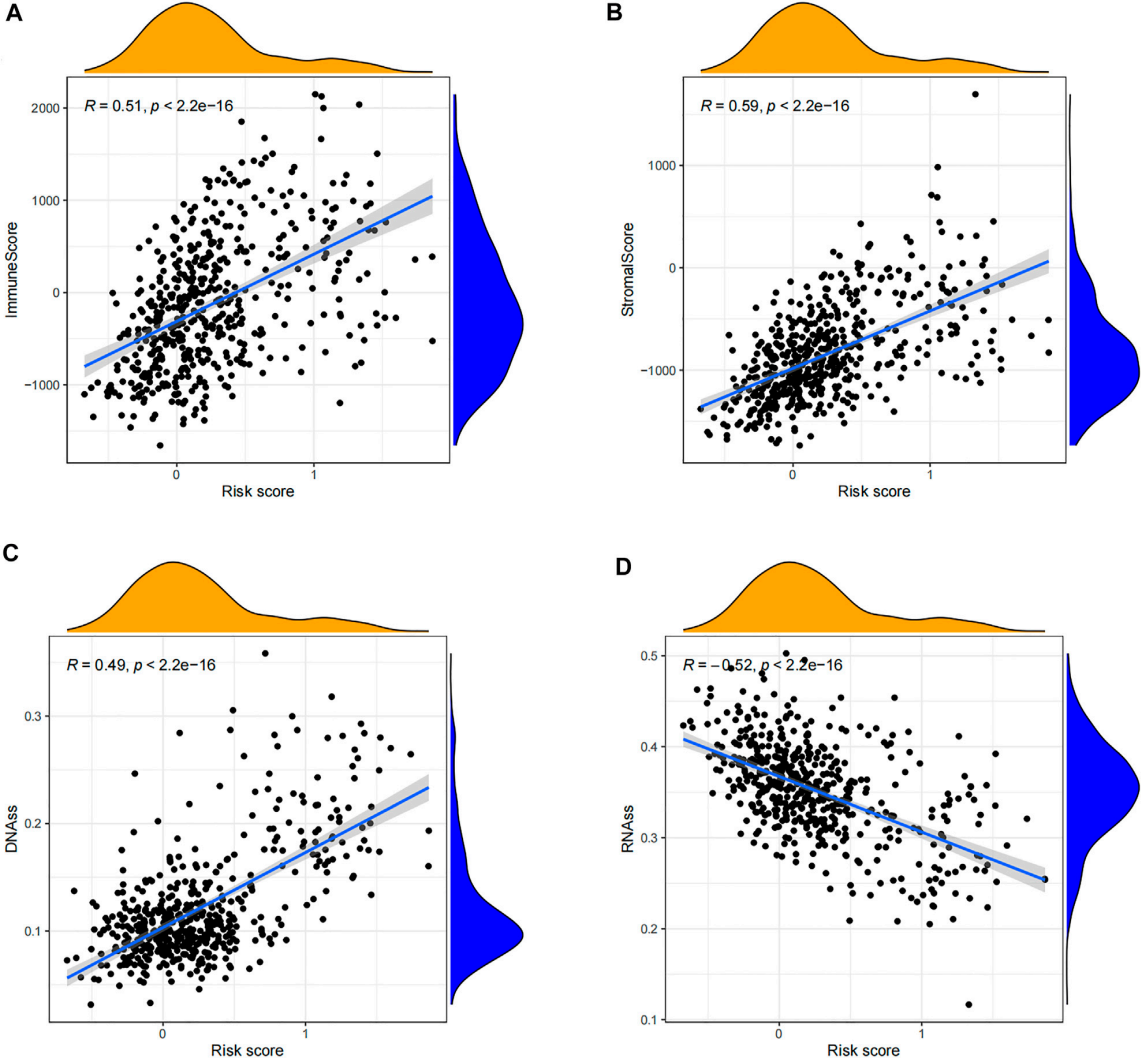
Correlation analysis of tumor microenvironment and stem cells. **(A)** Scatterplot of correlation between the immune cell score and risk score of LGG patients. **(B)** Scatterplot of correlation between the stromal cell score and risk score in LGG patients. **(C)** Scatterplot of the association between the stem cell score (DNAss) and risk score for LGG patients. **(D)** Scatterplot of the association between the stem cell score (RNAss) and risk score for LGG patients.

In order to clarify the impact of tumor microenvironment on the prognosis of LGG patients, we conducted a correlation analysis of the risk score and tumor microenvironment. From the scatterplot, it can be seen that both the immune score and the matrix score are significantly positively correlated with the patient’s risk score (*p* < 0.001) (Figures 6A,B), which indicates that the higher the content of immune cells and stromal cells in LGG patients, the higher the patient’s risk score. The greater the risk, the shorter the survival period. Next, the results of the stem cell correlation analysis showed that the risk score of LGG patients was significantly positively correlated with the stem cell score (DNAs) (*p* < 0.001), and was significantly negatively correlated with the stem cell score (RNAs) (*p* < 0.001) (Figures 6C,D). Therefore, the risk score of this prognostic model may be closely related to the activity of cancer stem cells.

### Drug Sensitivity Analysis

**FIGURE 7:**
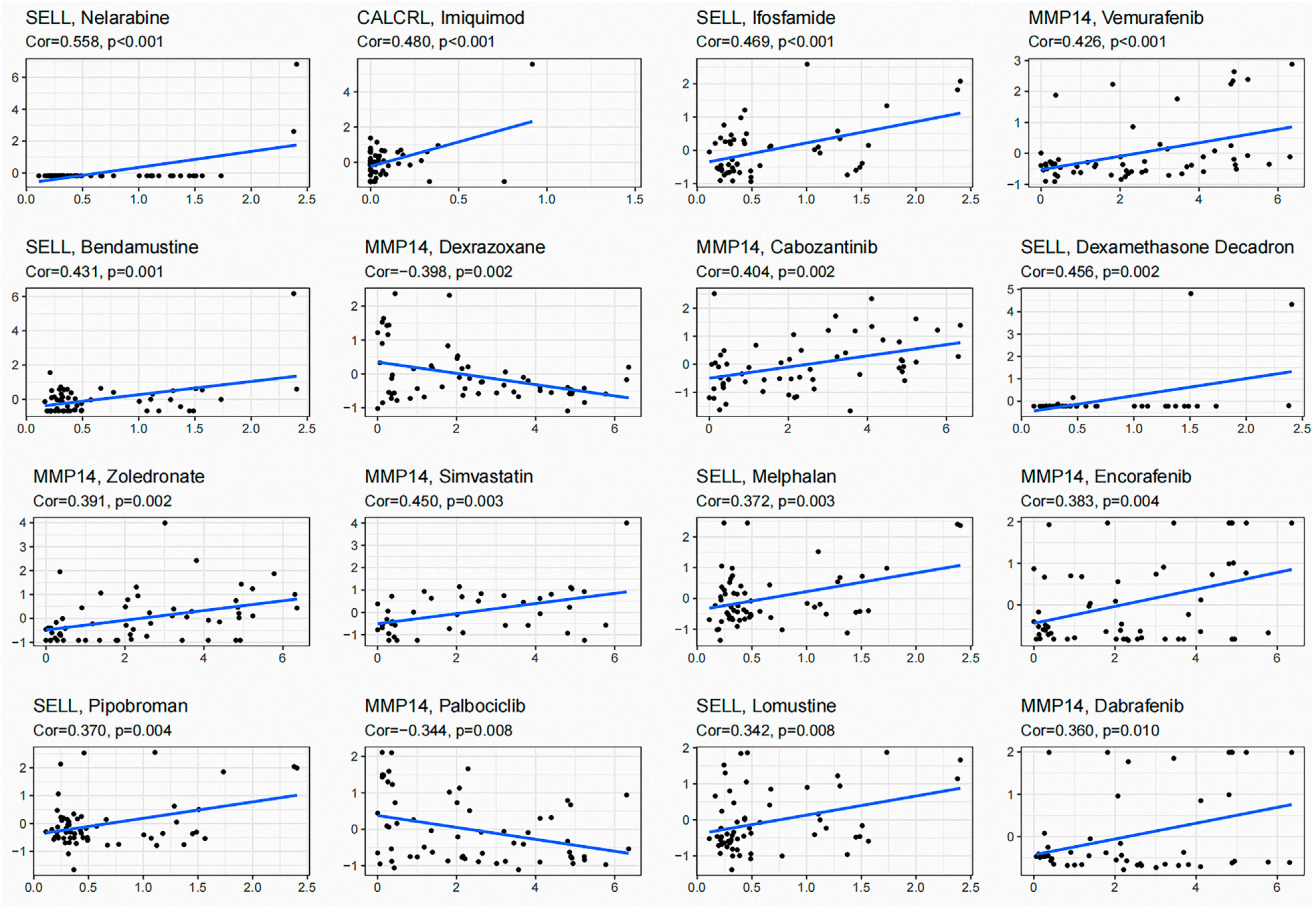
Gene–drug sensitivity analysis based on the CellMiner database; the top 16 drugs with high correlation with gene expression in inflammation-related prognosis models were screened.

**FIGURE 8:**
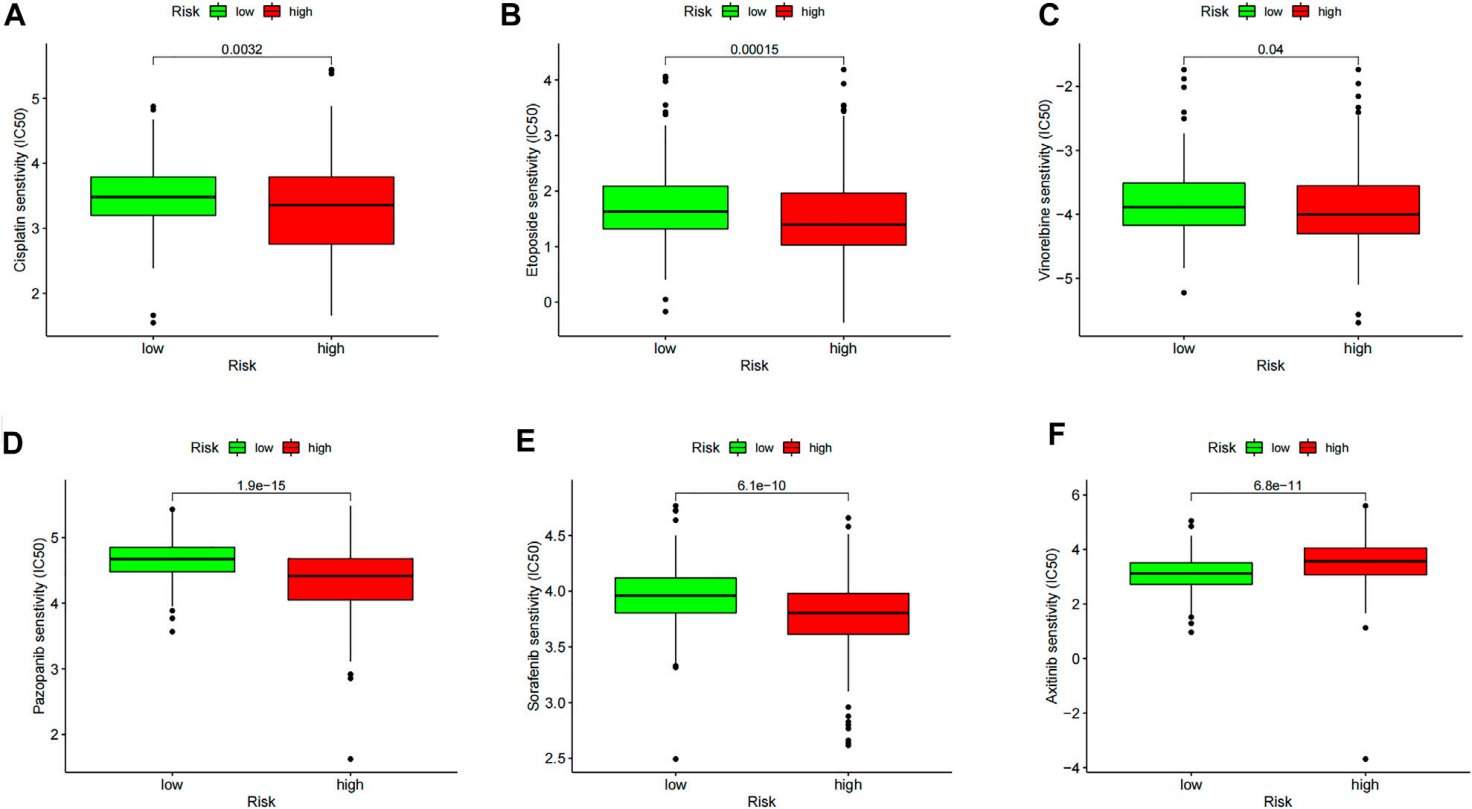
Model drug sensitivity analysis based on the “pRRophetic” R package; LGG therapeutic drugs related to inflammation-related prognosis models are screened. The drug sensitivity of **(A)** Cisplatin, **(B)** Etoposide, **(C)** Vinorelbine, **(D)** Pazopanib, **(E)** Sorafenib, and **(F)** Axitinib in High and Low Risk Groups respectively.

We obtained the top 16 drugs with the most statistically significant differences, by performing a separate drug sensitivity analysis on inflammation genes in the prognostic model. The results showed that the expression of SELL was positively correlated with the sensitivity of nelarabine, ifosfamide, bendamustine, dexamethasone Decadron, melphalan, pipobroman, and lomustine. It is indicating that the higher the expression of SELL, the stronger the sensitivity to the abovementioned drugs. The expression of MMP14 was positively correlated with the sensitivity of vemurafenib, cabozantinib, zoledronate, simvastatin, encorafenib, and dabrafenib, but it was negatively correlated with the sensitivity of dexrazoxane and palbociclib. In addition, the higher the expression of CALCRL in LGG patients, the patient’s sensitivity to imiquimod is stronger (Figure 7). In order to further improve the clinical value of inflammation-related prognosis models for the treatment of glioma, we analyzed the commonly used drugs in the clinical treatment of glioma, which include temozolomide, procarbazine, nitrosourea, vinblastine, podophyllotoxin, platinum, and molecular-targeted drugs targeting VEGF. The results showed that cisplatin, etoposide, vinorelbine, pazopanib, and sorafenib were more sensitive in the high-risk group than in the low-risk group, and axitinib was relatively weak in the high-risk group (*p* < 0.05) (Figures 8A–F, Supplementary Tables 3, 4, and 8).

## Discussion

The immortal proliferation of glioma cells continuously breaks the balance of the stable internal environment of normal brain cells and shapes the tumor microenvironment that is characterized by an immune-inflammatory response. In recent years, many studies have shown that some inflammatory cells (such as neutrophils and macrophages), inflammatory factors (IL-8, IL-1beta, IL-6, CD8^+^, and CXCL16) in the inflammatory microenvironment of glioma, and related signal pathways (NF-κB and STAT-3) are closely related to the progression and prognosis of glioma (Müller et al., 2017; Albrengues et al., 2018; Greten and Grivennikov, 2019; Zha et al., 2020). The inflammatory microenvironment can provide a good material preparation for tumor cell expansion and mutation, which makes the abnormally activated inflammatory response part of the reason why the overall survival rate of LGG is still low. Therefore, it is very meaningful to carry out in-depth research on the prognosis of LGG patients from the perspective of inflammation-related factors. Although the rapid development of high-throughput sequencing in recent years has brought new hope and direction for the accurate diagnosis and prognostic judgment of LGG (Kiran et al., 2019), so far, the number of biomarkers used to predict LGG in clinical practice is poor, which limits our early diagnosis and prediction of the therapeutic effect for LGG patients. It is shown that finding reliable and effective biomarkers is of great significance to prognosis prediction and clinical treatment of LGG. It has been confirmed that new biomarkers including glycoprotein YKL-40, microRNA, 21-mRNA, lncRNA, and BRAF gene mutations have significant relevance to the prognosis of LGG (Gandhi et al., 2018; Zhang et al., 2019; Song et al., 2020; Maimaiti et al., 2021; Gluexam et al., 2019; Zhang et al., 2020). In addition, serum biomarkers related to inflammation (neutrophil–lymphocyte ratio), adipokines, immune-related gene markers, autophagy-related genes (ARGs), energy metabolism genes, and repeated mutation genes (IDH, MGMT, EGFR, and chromosome 1p/19q deletion) are important prognostic factors for LGG (Chen et al., 2021; Zhou et al., 2018; Sun et al., 2014; Vachher et al., 2020; Wang et al., 2019; Ali et al., 2021; Zheng et al., 2018; Wu et al., 2019). However, these clinical pathologic and genetic factors are not specific, and may not achieve an accurate assessment of the survival rate of LGG. Therefore, it is important to conduct more comprehensive studies to increase the prognostic and predictive accuracy of the current assessment system. This study systematically analyzed the expression of 200 inflammation-related genes in LGG tissues and their relationship with the overall survival (OS).

Thirteen differentially expressed inflammatory genes were screened from the TCGA cohort, and after single-factor Cox analysis was conducted, nine inflammatory genes were observed to be related to LGG OS. Then, a prognosis model integrating three inflammatory response–related genes was finally constructed by LASSO regression analysis. According to the median risk score, patients were divided into the high-risk group and low-risk group. We found that the high-risk group was significantly associated with higher tumor grade, advanced TNM stage, and shorter OS stage. Independent prognostic analysis showed that the risk score was an independent predictor of OS.

The prognosis model established in this study consisted of three inflammatory response–related genes (CALCRL, MMP14, and SELL), which were upregulated in LGG tumor tissues. The CALCRL gene codes the calcitonin receptor–like receptor which is a seven-transmembrane domain G-protein–coupled receptor (Larrue et al., 2021 and Angenendt et al., 2019). Its expression products are closely related to CALCRL and RAMP expressed on the cell surface by co-expression with three receptor active modification proteins (RAMPs). CALCR can act as calcitonin gene–related peptide receptor 1 (CGRP1) when co-expressed with RAMP1, and when RAMP2 or RAMP3 exists, CALCR and its formed complexes can act as an adrenomedullin (ADM) receptor (Hay et al., 2011; Russell et al., 2014). CGRP is a neuropeptide which can promote tumor-related angiogenesis and tumor proliferation by regulating the signal transduction of the downstream molecule VEGF, which plays a key role in the occurrence and development of tumors (Toda et al., 2008). In addition to having the function of vasodilation to regulate blood pressure, ADM can be used as a hypoxia regulator to avoid the death of malignant tumor cells due to hypoxia and promote tumor cell growth (Russell et al., 2014). Therefore, CALCRL mediates the occurrence and development of tumor cells such as CGRP and ADM. Relevant studies have proved that the mRNAs of CALCRL/RAMP2 and CALCRL/RAMP3 are highly expressed in glioblastoma cell lines (Metellus et al., 2011). In addition, Benes L et al. have demonstrated that CRLR is widely distributed in human gliomas of different grades; at the same time, their team revealed that the possible mechanism of CRLR in gliomas is related to its influence on the formation of blood vessels, which assists the growth of gliomas (Ouafik et al., 2002; Benes et al., 2004). However, its mechanism of action in LGG is still unclear. Based on previous studies, we suspect that CRLR in low-grade glioma may boost the occurrence and development of LGG by influencing angiogenesis-related factors. Of course, this hypothesis still needs to be verified by experiment. MMP-14 is a subfamily of the matrix metalloproteinase family (MMPs). Studies have shown that MMP-14 can be used as a prognostic marker for patients with glioma (Wang et al., 2013). And some researchers found that the mechanism of MMP14 in glioma mainly works by cutting CD44 (Kajita et al., 2001), or *via* the combination of TIMP-2 and MMP14 to activate MMP-2 and MMP9 to enhance tumor invasion and tumor cell proliferation (Chernov et al., 2009). In addition, it also plays an important role in angiogenesis (Ulasov et al., 2014; Chen et al., 2016; Rooprai et al., 2016). Thus, MMP-14 is extremely important in predicting the prognosis of patients with glioma. The SELL gene is a gene encoding L-selectin with the smallest relative molecular mass in the selectin family. It is mainly distributed on the surface of white blood cells, endothelial cells, and platelets. SELL plays an extremely important physiological role in the development and metastasis of tumors. Tanriverdi et al. found that compared with low-grade glioma patients, selectins (E, L, and P-selectins), leukocyte adhesion molecules (ALCAM), and platelet endothelial cell adhesion molecules-1 (PECAM-1) are highly expressed in patients with high-grade gliomas. L-selectin pushes tumor plasma cell metastasis, which may be its main mechanism in glioma (Tanriverdi et al., 2017). In addition, recent evidence suggests that L-selectin may be an important target for cancer immunotherapy. Watson et al. discovered that in the immunotherapy of adoptive T-cell carcinoma in mouse models, L-selectin, which is overexpressed in T-cells, is related to the infiltration and enhanced proliferation of T-cells in tumors and controlling the growth of the tumor to a certain extent (Watson et al., 2019). In this study, our team found that SELL is differentially expressed in LGG, which indicates that it may be closely related to the degree of immune cell infiltration in tumors.

With deeper understanding of the relationship between inflammation and tumors, in recent years, studies have found that the relationship between inflammation and the immune system of tumor cells cannot be ignored. Zong Z et al. found that the inflammatory cytokine IL-1β in liver cancer can induce PD-L1 expression through the transcription factors p65 and IRF1, which creates opportunities for tumor cells to escape the immune system (Zong et al., 2019). Zhang W et al. reported that the upregulated IL-6 in liver cancer can downregulate the O-type protein tyrosine phosphatase receptor (PTPRO) by activating JAK2/STAT3 signal transduction, resulting in high PD-L1 expression, inducing immunosuppression and promoting tumor growth (Zhang et al., 2020). These studies show that when a powerful foreign factor invades, the body needs to activate a stronger defense system–immune response, and activated immune cells will produce inflammatory factors, which can pass immune- and inflammation-related cell signaling pathways to induce tumor cells to highly express immunosuppressive signal molecules, and further induce the occurrence of tumors. This study investigated the inflammation-related risk model to predict the immunotherapy response and found that the expression of PD-1, PD-L1, and CTLA4 was significantly upregulated in the high-risk group, and the expression and risk score of PD-1, PD-L1, and CTLA4 are positively correlated. Therefore, the abovementioned results indicate that patients in the high-risk group can benefit from immunotherapy more clinically. Besides, Berghoff AS et al. reported that the pro-inflammatory cytokine IFN-γ can drive the high expression of PD-L1 in tumor cells (Berghoff et al., 2015). Bloch O et al. found that glioma promotes immune suppression by regulating IL-10 signal transduction and then upregulating the expression of B7-H1 in tumor infiltration–related macrophages, resulting in high PD-L1 expression (Bloch et al., 2013). Liu et al., 2019 showed that inflammatory factors can upregulate the expression of PD-L1/PD-1 in tumor cells to assist tumor cell immune escape and promote tumor cell proliferation (Liu et al., 2020). On these bases, our research found that based on GSEA analysis, tumor-related signaling pathways such as antigen processing and presentation, primary immunodeficiency, natural killer cell–mediated cytotoxicity, intestinal immune IGA production network, B-cell receptor signaling pathway, and T-cell receptor 64 pathways such as body signaling pathways are significantly abundant in the high-risk group, and the continuous activation of these pathways has been confirmed to be related to LGG. A total of 13 immune-related functional pathways, including APC co-inhibition, APC co-stimulation, check point, and T-cell co-inhibition, were more significant in the high-risk group; B-cells, CD8^+^ T-cell, iDCs, pDCs, macrophages, neutrophils, T-helper cells, Tfh, Th1 cells, Th2 cells, TIL, Tregs, and other immune cell subsets were significantly enriched in the high-risk group, further verifying that inflammation is closely related to tumor progression. The increased activity of Tfh, Th1 cells, Th2 cells, TIL, and Tregs in the high-risk group indicates that the immune regulation function of the high-risk group is disturbed, and the antitumor immunity is weakened. In addition, the proportion of macrophages, neutrophils, and Treg cells is higher in the high-risk group, indicating that their increase can promote immune invasion, which is closely related to the poor prognosis of LGG patients. In order to gain a deeper understanding of the relationship between risk scores and immune components, we examined the role of risk scores in the types of immune infiltration. We found that the high-risk score was significantly correlated with C3, whereas the low-risk score was correlated with C5, indicating that C3 promotes the occurrence and development of tumors. This finding is consistent with the results of previous studies. By blocking the PD-1/PD-L1 pathway, immune checkpoint inhibitors can relieve the tumor microenvironment’s inhibitory effect on immune cells and activate the body’s immune function to achieve antitumor effects. Immunotherapy based on immune checkpoint inhibitors has been widely used in other types of tumors. Although, so far, immunotherapy has not been approved for glioma treatment, some preclinical studies have shown its therapeutic potential. For example, a randomized trial was conducted in 35 patients with recurrent glioblastoma (GBM). The survival time of the neoadjuvant pembrolizumab group was longer than that of the adjuvant pembrolizumab group (median PFS was 2.4 and 3.3 months, and median OS was 13.7 and 7.5 months, respectively). The trials have shown the therapeutic potential of immunotherapy in the neoadjuvant treatment of GBM (Cloughesy et al., 2019). A phase II clinical trial (NCT03890952) is currently underway to evaluate PD-L1 and other immune biomarkers. It mainly compares the combination of nivolumab and bevacizumab in patients who have not undergone surgery and those undergoing surgery. It is expected that nivolumab can be obtained from recurrent GBM (Zimmer et al., 2020). Also, Hideho Okada et al. used glioma-associated antigen (GAA) immune-vaccine therapy to target and activate cytotoxic T cells (CTL) on the cell surface to treat LGG (Sanders and Debinski, 2020). Studies have confirmed GAA. It has strong specificity and good tolerance, and can effectively control the occurrence and development of LGG. Although most of the current clinical studies are mainly targeting glioblastoma with a high degree of malignancy, there are few clinical trials of immunotherapy in LGG patients, but a relatively high specific immune checkpoint has been found to effectively screen the benefits of immunotherapy. The population will be of great significance in the treatment of LGG patients and the prevention of malignant transformation and recurrence of low-grade gliomas.

It has been confirmed that inflammatory cytokines can induce epithelial–mesenchymal cell transformation (EMT) and cancer stem cell (CSC) production and related molecular regulation to establish an inseparable connection with the tumor microenvironment (Markopoulos et al., 2019). Cancer stem cells (CSCs) are often referred to as tumor-initiating cells. Due to their self-renewal ability and heterogeneity, they are the main cause of tumor resistance, recurrence, and metastasis (Scheel and Weinberg, 2012). In our study, the correlation between the prognostic gene expression and cancer stem cell score suggests that the risk score of the model composed of CALCRL, MMP14, and SELL is significantly positively correlated with the stem cell score (DNAss), suggesting that the risk of this prognostic model may be closely related to the activity of cancer stem cells. At the same time, this study clarifies that the increased expression of SELL is related to elevation in the sensitivity of cancer cells to lomustine. Lomustine is a drug containing a classical chemotherapy regimen in the guide for WHO grade II gliomas PCR regimen (promethazine + lomustine + Changchun Neobase) (Shaw et al., 2012). Therefore, the high expression of the SELL gene can predict the sensitivity of LGG patients to this drug. The expression of MMP14 is positively correlated with the sensitivity of vemurafenib. Vemurafenib is a competitive small-molecule BRAF V600E inhibitor that can act on BRAF V600E mutations in low-grade gliomas. It has been proven to be effective in treating metastasis melanoma which is prone to BRAF mutation. However, recent clinical trials have shown that the drug has good efficacy in BRAF V600E mutant malignant astrocytomas and low-grade gliomas; patients with high expression of MMP14 may predict the better curative effect of vemurafenib treatment (Del et al., 2018; Van et al., 2018). Therefore, the genes in our prognostic model can be used as targets to predict drug sensitivity.

In summary, our study has determined a new prognostic marker model composed of three inflammatory response–related genes. In the TCGC database, this model has been proven to be independently related to OS, and it has been proven to be of great significance in regulating the immune microenvironment, tumor microenvironment, and drug sensitivity. It provides a novel idea and method for LGG prognosis, immunotherapy, and drug sensitivity evaluation. However, the specific underlying mechanism between LGG inflammation–related genes and tumor immunity is still unclear, and further research is needed.

## Conclusion

The inflammatory gene–related prediction model proposed in this study is of great significance for the screening of prognostic markers in LGG patients, especially in the exploration of immune response, tumor microenvironment, and immunotherapy sensitivity. It is expected to be the basic research and immunotherapy of LGG immunity. The choice of method provides an important reference and clinical transformation value.

## Data Availability

The datasets presented in this study can be found in online repositories. The names of the repository/repositories and accession number(s) can be found in the article/Supplementary Material.
